# A historical overview on the rise and downfall of psychosurgery

**DOI:** 10.1192/j.eurpsy.2025.2130

**Published:** 2025-08-26

**Authors:** G. N. Porfyri, V. Tarantili

**Affiliations:** 1 National and Kapodistrian University of Athens, Athens; 2 General Hospital of Argos, Argos, Greece

## Abstract

**Introduction:**

“Psychosurgery” is defined as the human brain surgery to treat psychiatric symptoms.

**Objectives:**

This study aims to portray psychosurgery’s historical evolution.

**Methods:**

A review of 35 articles from 2000 to 2024 on PubMed and Google Scholar, regarding psychosurgery.

**Results:**

The initial phase of psychosurgery dated in 1888, when Swiss psychiatrist Gottlieb Burckhardt, in en effort to control the symptoms of psychiatric patients, he performed the very first brain topectomies. Later on, in 1936, Portuguese neurologist Egas Moniz and neurosurgeon Almeida Lima, collaborated on performing the first lobotomy. After achieving 20 lobotomies, Moniz reported that 35% of patients showed complete remission of psychiatric symptoms, 35% of patients showed a mild improvement, and 30% of patients showed no improvement. It was Freeman’s (a neurologist) and Watts’s (a neurosurgeon) turn to perform in 1936 the first lobotomy on the USA ground, on a woman suffering from depression; Alice Hammatt. Unfortunately, six days post-operation, Hammatt experienced language difficulties, disorientation and agitation. However, the surgery was still considered a success. By 1942, Freeman and Watts had performed 200 lobotomies, declaring that 63% of patients improved, 23% of patients showed no relief, while 14% of patients suffered complications, including death. Freeman pursued with the development of a transorbital method which he tended to perform in outpatient departments, without any neurosurgical assistance, having patients anesthesized with a portable electroshock machine. Profoundly disapproving this method, Watts, decided to end their partnership. Further unsatisfactory outcomes like the lobotomy of Rosemary Kennedy (sister of President John F. Kennedy) shaped an additional negative image of Freeman’s work. Rosemary who initially was subjected to lobotomy due to mild developmental delays, anxiety and epilepsy, was postoperatively left severely disabled, without autonomy, being institutionalized for the rest of her life. Finally, the approval of chlorpromazine’s use in the US (1955) alongside public awareness over psychosurgery’s complications, such as the “post-leucotomy syndrome” causing disinhibition as well as “lobotomy criteria” which included female gender, non-obeidance, hospitalization in overcrowed institutions, opposite political opinion, leaded to psychosurgery’s dowfall.

**Conclusions:**

Considering the dark history of psychosurgery, it is imperative to proceed to such treatments exclusively to diseases with a well decoded neurophysiology, always respecting human rights and protecting patients’ dignity and self-will.

**Disclosure of Interest:**

None Declared

